# Aerobic training for improved memory in patients with stress-related exhaustion: a randomized controlled trial

**DOI:** 10.1186/s12888-017-1457-1

**Published:** 2017-09-02

**Authors:** Therese Eskilsson, Lisbeth Slunga Järvholm, Hanna Malmberg Gavelin, Anna Stigsdotter Neely, Carl-Johan Boraxbekk

**Affiliations:** 1 Department of Community Medicine and Rehabilitation, Physiotherapy, Umeå University, SE- 901 87 Umeå, Sweden; 2 Department of Public Health and Clinical Medicine, Occupational and Environmental Medicine, Umeå University, Umeå, Sweden; 3 Department of Psychology, Umeå University, Umeå, Sweden; 4 Department of Social and Psychological Studies, Karlstad University, Karlstad, Sweden; 5 Danish Research Centre for Magnetic Resonance (DRCMR), Centre for Functional and Diagnostic Imaging and Research, Copenhagen University Hospital Hvidovre, Copenhagen, Denmark; 6 Center for Demographic and Aging Research (CEDAR), Umeå University, Umeå, Sweden; 7 Umeå Centre for Functional Brain Imaging (UFBI), Umeå University, Umeå, Sweden

**Keywords:** Cognition, Episodic memory, Exercise, Burnout, Anxiety, Depression

## Abstract

**Background:**

Patients with stress-related exhaustion suffer from cognitive impairments, which often remain after psychological treatment or work place interventions. It is important to find effective treatments that can address this problem. Therefore, the aim of this study was to investigate the effects on cognitive performance and psychological variables of a 12-week aerobic training program performed at a moderate-vigorous intensity for patients with exhaustion disorder who participated in a multimodal rehabilitation program.

**Methods:**

In this open-label, parallel, randomized and controlled trial, 88 patients diagnosed with exhaustion disorder participated in a 24-week multimodal rehabilitation program. After 12 weeks in the program the patients were randomized to either a 12-week aerobic training intervention or to a control group with no additional training. Primary outcome measure was cognitive function, and secondary outcome measures were psychological health variables and aerobic capacity.

**Results:**

In total, 51% patients in the aerobic training group and 78% patients in the control group completed the intervention period. The aerobic training group significantly improved in maximal oxygen uptake and episodic memory performance. No additional improvement in burnout, depression or anxiety was observed in the aerobic group compared with controls.

**Conclusion:**

Aerobic training at a moderate-vigorous intensity within a multimodal rehabilitation program for patients with exhaustion disorder facilitated episodic memory. A future challenge would be the clinical implementation of aerobic training and methods to increase feasibility in this patient group.

**Trial registration:**

ClinicalTrials.gov: NCT03073772. Retrospectively registered 21 February 2017.

## Background

Mental disorders are the most common reasons for sick leave in Sweden, and exhaustion disorder (ED) is one of the most common diagnoses leading to reduced work ability [1]. ED is characterized by pronounced physical and mental exhaustion during at least two weeks, with identifiable stressors (work- or non-work-related) present for at least 6 months [2]. The Swedish National Board of Health and Welfare presented clinical diagnostic criteria for ED in 2003 [3]. Memory and concentration problems are included in the diagnostic criteria and have been manifested as deficiencies in executive functions, attention, episodic and working memory [2]. ED patients also frequently report high levels of mental symptoms such as burnout, depression and anxiety [4], as well as various somatic symptoms such as nausea, headaches and dizziness [5]. A common method of rehabilitation is multimodal rehabilitation (MMR) containing components of group-based or individual cognitive behavioural therapy (CBT), physical activities and work training coordinated by an interdisciplinary team. Unfortunately, rehabilitation may be difficult and patients often still report mental symptoms [4], somatic symptoms [5] and reduced work ability [6], even after extensive MMR. Further, cognitive impairments may also remain after individual psychological treatment in this patient group [7] as well as after work place interventions [8]. We recently showed that a 12-week process-based cognitive training intervention for patients with ED significantly improved performance on the executive function updating and episodic memory, as well as reducing subjective memory complaints and the level of burnout in comparison to a control group [9]. These findings highlight the need for specifically targeting cognitive functions during rehabilitation.

Another known interventional approach to improve cognitive functions is aerobic exercise [10, 11]. Converging evidence shows that physical activity strengthens brain-behaviour relationships throughout the lifespan [12], and specifically performance on executive functions have shown to be improved following increased aerobic capacity [10]. Notably, aerobic training interventions have also been found to effectively reduce symptoms of depression [13]. Hence, aerobic training may be of particular interest for ED patients considering that both executive failure and depressive symptoms are present in this patient group.

To our knowledge, only one study has examined the effect on cognition after a 12-week aerobic training program at a moderate intensity in participants with occupational burnout. In that particular study, the aerobic training was supervised and the participants were allowed to use varying equipments for cardiovascular training at a fitness center. After the intervention, the participants, all males, significantly improved executive function performance to similar levels as healthy controls [14]. Notably, however, clinical populations with ED have an over-representation of women, involve patients that are on sick leave, and patients taking antidepressants [5]. Thus, more studies regarding the potential of aerobic training in rehabilitation of patients with ED are needed, and in particular randomized controlled trials.

The aim of the present study was to investigate effects on cognitive performance of a 12-week aerobic training program performed at a moderate-vigorous intensity and added to a MMR program. All participants in the study were patients with ED who participated in the same MMR program. The intervention group was compared to the patient group receiving only regular MMR. We hypothesised that aerobic training would lead to a general facilitation of cognitive performance accompanied with reduced levels of burnout, depression and anxiety.

## Methods

### Study design and participants

This study was a 12-week intervention study using an open-label, parallel and randomized controlled design. The study is part of a larger randomized controlled study for patients with ED which includes a one-year follow-up, Rehabilitation for Improved Cognition (RECO). RECO was performed at the Stress Rehabilitation Clinic, University Hospital of Umeå, from April 2010 until June 2013. A total of 272 patients were consecutively screened for eligibility in the RECO study. In order to speed up the recruitment process, eight participants were recruited directly from the Social Insurance Agency in Umeå, Sweden. Inclusion criteria in the study were: confirmed ED; 18-60 years of age; current employment; considered by a physician and a psychologist as suitable for group-based MMR; not in the need of other treatment or rehabilitation; no known abuse of alcohol or drugs; not currently participating in any other interventional study. All patients were on sick leave because of ED.

In total 231 patients fulfilled the inclusion criteria of the RECO study and 161 of these patients accepted to participate. They received verbal and written information about the study and provided written informed consent before inclusion. During the first 12 weeks of MMR there were another 29 patients who declined to participate, which finally resulted in 132 patients who were randomized prior to the start of the intervention. The participants were enrolled by the responsible physician and the project coordinator, and were assigned to the intervention by the project coordinator. Forty-seven patients were allocated to MMR with additional aerobic training and 41 patients were allocated to MMR with no additional training (control group). The remaining 44 patients were allocated to MMR with additional cognitive training, which has been described previously [9]. This study was approved by the Regional Ethical Review Board in Umeå, Sweden (Approval Nr. 2010-53-31) and was conducted in accordance with the ethical principles of the Declaration of Helsinki. The CONSORT 2010 guidelines were used to describe this randomized controlled trial.

### Procedure

**Fig. 1:**
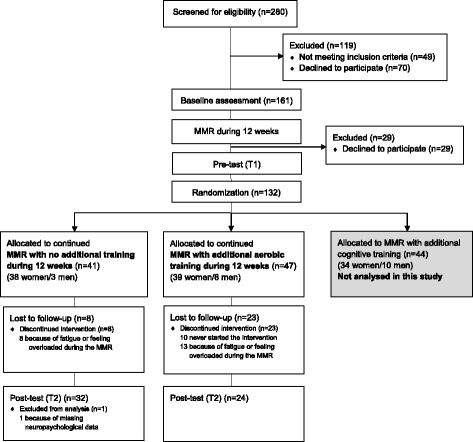
CONSORT flow diagram

All patients in the RECO study participated at the Stress Rehabilitation Clinic in a 24-week MMR, which consisted of group-based cognitive behavioural therapy (CBT), vocational measures with rehabilitation meetings, and individual physical activity on prescription (PAP) [9]. A physiotherapist met the patient in a one-hour patient-centred counselling and prescribed an individualized written PAP in accordance with the scientific knowledge bank “Physical Activity in the Prevention and Treatment of Disease” (FYSS). The goal was to provide support to the patient to reach the recommendations of physical activity, i.e., at least 150 min of moderate intensity exercise every week, preferably 3-5 days per week [15]. Four and 12-week follow-up of the PAP was performed by phone. Each CBT group comprised eight patients and met weekly in 22 three-hour sessions. Each patient also received two individual meetings to determine and evaluate individual targets for behavioural change. After 12 weeks of MMR, a person who was independent to the project conducted a randomization by CBT group by drawing lots with a 1:1:1 allocation into one of three conditions; (1) continued MMR with no additional training, (2) continued MMR with an addition of aerobic training, and (3) continued MMR with an addition of computerized process-based cognitive training. The lots were prepared by the project coordinator. They were stored safely and the content of a single lot was not revealed until after having been drawn. The training intervention was performed during the last 12 weeks of the MMR. Because of high dropout rates in the aerobic training group and the cognitive training group, the randomization was adjusted in the latter part of the study with a doubled possibility to receive one of the training programs compared to the regular MMR group with no additional training. Due to the open-label design, neither patients, health care providers nor data collectors were blinded to patients´ group assignment. A flowchart of patients through the trial with information on reasons for dropping out is illustrated in Fig. 1. All participants in the study received a compensation of 600 SEK.

### Intervention

The aerobic training was performed as group indoor cycling (“spinning”), at training centres in the municipality conveniently located for the patient. Each training session was 40 min long, and performed three times each week for 12 weeks, giving a total of 36 training sessions. Heart rate was monitored during all sessions using a chest belt heart monitor (Polar RS800). The patients were instructed to exercise at a moderate-vigorous intensity, a load within 70-85% of their maximum age-adjusted heart rate (220 – age). Data from the aerobic exercise training was stored and analysed (Polar ProTrainer) every week by a physiotherapist at the Stress Rehabilitation Clinic. Each week the physiotherapist provided written feedback to the patients about appropriate training intensity as well as encouraging comments about their training.

### Measurements

In RECO, baseline characteristics were assessed before starting the MMR (baseline). The primary outcome was cognitive function and secondary outcomes were psychological variables and aerobic capacity. These outcome measures were assessed before randomization at week 12 of the MMR (T1) and after the intervention period at week 24 (T2). During the intervention period, the patients registered their overall physical activity in a diary including daily walks (low-moderate intensity for at least 30 min each time). A physiotherapist instructed them how to rate the level of intensity by using Borg’s Rating of Perceived Exertion (RPE) scale (6-20 points).

#### Baseline characteristics

Sex, age, educational level, marital status, family situation and sick leave before MMR were assessed by questionnaires. Body mass index (BMI) kg/m^2^ was recorded and calculated in conjunction with the submaximal test. Estimated total physical activity during a normal week was assessed by self-administrated questions about “every-day” physical activity (e.g. cycling, walks, heavier housework, snow shovelling, gardening), which was dichotomized into <30 min per day and ≥30 min per day, and physical exercise (e.g. gymnastics, running, soccer), which was dichotomized into ≤2 h per week or >2 h per week. Verbal ability was assessed with SRB:1, a multiple-choice synonym test [16].

#### Aerobic capacity

To estimate maximal oxygen uptake (VO_2max_) we used a submaximal test on a calibrated cycle ergometer (Monark Ergomedic 828E). The cycle resistance was adjusted in order to reach a steady state in heart rate of at least 120 beats per minutes with the cadence of 50 rpm. Each minute the heart rate was registered with Polar RS800, and the perceived exertion was registered using Borg’s Rating of Perceived Exertion (RPE) scale (6-20 points). The mean heart rate at the last three minutes of the test was recorded as the steady state heart rate. VO_2max_ (ml/kg/min) was estimated by Åstrands nomogram, adjusted for sex, years of age, body weight, workload and steady state heart rate [17].

Heart rate recovery (HRR) was an exploratory outcome variable, which we used to assess cardiac autonomic regulation. It was measured immediately after the submaximal test where the patient was asked to sit down in a chair for recovery. The difference between the last heart rate value during the submaximal test and the heart rate after one minute of recovery was defined as the HRR.

#### Cognitive function

Cognitive functions were assessed using a test battery covering five cognitive domains: executive function; working memory; episodic memory; perceptual speed; and reasoning ability. The cognitive test battery used in this study has previously been described in detail [9]. In short:

*Executive function*: *Letter memory running span* was used as a measure of updating [18]. In this task, lists of single letters were presented serially on a computer screen and patients were asked to recall the four last presented letters in the correct order. Another measure of updating was the *n-back task*, in which patients were presented with lists of digits (1-9) and asked to indicate whether each number matched the number presented one, two or three positions previously [19]. Accuracy in the 3-back condition was used as outcome measure. Inhibition was assessed using the Colour word interference test (also known as the Stroop test) from D-KEFS [20]. The Trail making test, also from D-KEFS, was used as a measure of attentional shifting [20]. Both of these tasks were administered according to standard procedures and performance was measured in time (seconds) to complete the task, with faster times indicating better performance.

*Working memory:* In *Digit span* (WAIS-R) participants were instructed to recall digits forwards and backwards [21]. *Letter-number sequencing* was adapted from WAIS-III [22], requiring patients to recall numbers and letters according to numerical and alphabetical order. These tests were administered according to standard procedures and performance was measured as number of correctly recalled sequences.

*Episodic memory: Recall of concrete nouns* was used as a measure of episodic memory. A list of 18 concrete nouns was administered as a multiple-trial free-recall task according to Buschke’s selective reminding procedure [23] and total number of words recalled on consecutive trials without reminding.

*Perceptual speed: Digit symbol* from WAIS-R [21] was used to assess perceptual speed, requiring patients to draw geometric symbols into empty boxes, according to a coding key. Number of correct symbols completed in 90 s was used as outcome measure.

*Reasoning ability: Raven’s Advanced Progressive Matrices* [24] was used to assess non-verbal reasoning ability. The test consists of 36 different pattern matrices and was split into two parts using odd and even items. The number of correctly completed matrices in 10 min was used as outcome measure.

#### Psychological variables

Burnout was quantified with the Shirom-Melamed Burnout Questionnaire (SMBQ) [25], which contains 22 items, each rated on a 7-point scale (1-7). An overall index was computed as the mean of all items, where a higher score indicates a higher level of burnout. The SMBQ is a valid and reliable instrument [26] and in this present sample the Cronbach’s alpha was .90. Anxiety and depression was measured with the Hospital Anxiety and Depression Scale (HAD), consisting of 14 items divided into two subscales. Both subscales consist of 7 items scored on a 4-point scale (0-3). The composite total score (0-21 points) was calculated and a higher score indicate more symptoms [27]. The HAD instrument has been shown to be a valid instrument [28] and in this present sample the Cronbach’s alpha was .84 for anxiety and .86 for depression.

#### Structural magnetic resonance imaging (sMRI)

When designing this study, we also aimed at investigating in an exploratory approach the relationship between improved fitness and structural brain changes. Unfortunately, the group with exercise participants that underwent MRI at pre-test suffered from large attrition. For only six of the participants in the aerobic training group and 12 in the control group we had complete sMRI pre-and post-training. The parameters for T1 images were: 180 slices; 1 mm thickness; TR 8.2 ms; TE 3.2 ms; flip angle 12°; field of view 25 × 25 cm, acquired with a 3 T General Electric scanner equipped with a 32-channel head coil. Even though this is a limited sample size we decided, for explorative purposes, to examine whether these participants showed any indications of a general increase in hippocampus volume, a brain structure that has been shown to be specifically sensitive to improved oxygen uptake following aerobic exercise [29, 30]. We estimated hippocampus volume using Freesurfer [31], version 6-beta (20151015) longitudinal stream [32]. We combined left and right hippocampus volume.

### Statistics

Statistical analyses were performed using IBM SPSS Statistics version 22 (SPSS Inc., Chicago, IL, USA). A power calculation was performed based on results from a previous pilot study on patients with ED participating in a computerized process-based cognitive training. They improved in performance in the cognitive task letter memory running span from 3.0 (1.4) pre-test to 5.8 (1.9) post-test. Based on these results we estimated a need of approximately 30 patients in each group to reach 80% power to detect a statistically significant difference (*p* < 0.05) between the groups. All analyses were based on per-protocol analysis where only patients who completed the 12-week intervention period were included. Differences were considered statistically significant at *p* < 0.05.

Pearson’s Chi^2^ -tests (categorical variables) and independent samples *t*-tests (continuous variables) were used for between-group analyses of baseline characteristics and subgroup analyses. In the present study, we were interested in examining the effects at the cognitive ability level rather than at the single test level. Such an approach has been emphasized as missing in previous interventional studies despite being of greater validity when trying to understand how exercise may affect cognitive performance [31]. Therefore, we computed domain-specific cognitive scores by first converting all test results to z scores. Z scores for respective test at each time point (T1 and T2) were standardized to the baseline mean and SD: ZT1_ind_ = (XT1_ind_ – MT1)/SDT1; ZT2_ind_ = (XT2_ind_ – MT1)/SDT1. Then we computed an average z score of each cognitive domain. In addition, a global cognitive function score was calculated as the mean z scores of all domains. To investigate changes over time in aerobic capacity, cognitive function, psychological variables, Group x Time repeated measures ANOVAs were performed. η_p_^2^ values ranging .010 to < .059 were interpreted as small effects, .059 to < .138 as medium effects, and .138 or more as large effects [33]. When significant group by time interaction was found a paired sample *t*-test was used to investigate change between T1 to T2 in each group, respectively.

For each significant group by time interaction we planned to further examine the association between improved aerobic capacity and improved cognitive performance by correlating (Pearsons) change in aerobic capacity and change in cognitive performance (T2-T1). We also planned to investigate subgroups of individuals in the aerobic training group who improved or not in a specific cognitive domain. The dichotomization was performed on the median value.

An expectation-maximization method was performed for imputation to adjust for missing responses in single items. For the cognitive function, this was done for three participants with missing single test results. Missing responses in single items in variables of burnout, depression and anxiety occurred in 0.17–0.59% of the items. If responses to more than three items were missing in burnout and more than one missing in depression and anxiety, respectively, the participant was excluded from the analysis.

## Results

A total of 24 patients in the aerobic training group and 32 in the control group completed the 12-week intervention period. Characteristics for the two groups at baseline are presented in Table 1. There were no significant differences between the groups at the start of the study.

**Table 1:** Characteristics at baseline of the study population

Variable	Aerobic training group (*n* = 24)	Control group (*n* = 32)	*P*-value
Sex, female/male, *n*	22/2	30/2	1.000
Age mean (SD), years	42.00 (8.61)	41.69 (7.88)	0.888
Education, *n* (%)			
University	17 (71)	20 (62)	0.578
Marital status, *n* (%)			
Married/co-habited	18 (75)	24 (75)	0.544
Family situation, *n* (%)			
Living with children at home	19 (79)	25 (78)	1.000
Sick leave before MMR, *n* (%)			
No sick leave^a^	1 (4)	4 (13)	
≤6 month	17 (71)	22 (69)	
7-12 months	2 (8)	3 (9)	
>12 months	4 (17)	3 (9)	0.739
BMI kg/m^2^, mean (SD)	24.87 (4.69)	25.92 (4.94)	0.424
Physical activity, *n* (%)			
<30 min/day	8 (33)	13 (41)	
≥30 min/day	16 (67)	19 (59)	0.781
Physical exercise, *n* (%)			
≤2 h/week	20 (83)	27 (84)	
>2 h/week	4 (17)	5 (16)	1.000
Verbal ability, mean (SD)	23.38 (3.50)	23.50 (3.51)	0.895

*MMR* Multimodal rehabilitation, *BMI* Body Mass Index, *SD* standard deviation

^a^these patients had earlier been on long-term sick leave because of exhaustion disorder, but were not entitled to sickness benefit at this time

In total, there were 32 patients (25 women and 7 men) with a mean age of 45.31 (SD 7.7) years who were lost to follow-up. Significantly more drop-outs were presented in the aerobic training group (49%) compared to the control group (22%) (*p* = 0.01). It should be noted, however, that 10 patients in the aerobic training group dropped out before the start of the intervention. A common reason for dropping-out was that the ordinary MMR was challenging enough and that further intervention was perceived too much effort (see Fig. 1). In baseline characteristics, there was only a significant difference in BMI between the patients who completed the intervention period (mean value 25.5, SD 4.8) and the drop-outs (mean value 28.4, SD 5.1) (*p* = 0.01).

During the intervention period, there was no significant difference between the groups in self-registered number of walks. The aerobic training group reported 38.0 (SD 22.8) walks and the control group reported 38.3 (SD 24.9) walks. Patients in the aerobic training group attended on average 20.7 (SD 8.7) of 36 planned training sessions (58%). On average, 77% of each training session was performed at a moderate-vigorous intensity.

### Aerobic capacity

A significant time by group interaction was demonstrated, where the aerobic training group significantly improved in VO_2max_ more so than the controls. Aerobic capacity increased by 8.8% for the aerobic training group and 0.6% for the control group. No significant time by group interaction effect was observed in HRR (Table 2).

**Table 2:** Group means and standard deviations based on repeated measures ANOVAs in aerobic capacity and self-reported psychological variables, in the aerobic training group and the control group before and after the intervention

Variable	Aerobic training group (*n* = 24)		Control group (*n* = 32)		Group x Time		
	T1	T2	T1	T2	F	*p-value*	η_p_^2^
Aerobic capacity							
VO_2max_^a^ (ml/kg/min)	36.91 (7.31)	40.48 (9.78)	36.61 (9.83)	36.83 (10.55)	F(1,53) = 5.01	0.03	0.09
Heart rate recovery^b^ (beats/min)	39.26 (11.21)	44.52 (8.94)	38.23 (8.02)	39.77 (9.88)	F(1.51) = 2.75	0.10	0.05
Psychological variables							
Burnout^c^	4.75 (0.98)	3.92 (1.10)	4.84 (1.01)	4.40 (1.08)	F(1,54) = 2.82	0.10	0.05
Anxiety^d^	9.50 (3.92)	7.50 (3.12)	9.71 (3.87)	8.06 (3.65)	F(1,54) = 0.18	0.67	0.00
Depression^d^	6.46 (4.10)	4.46 (3.79)	7.47 (3.61)	6.16 (4.04)	F(1,54) = 0.71	0.40	0.01

^a)^Aerobic training group, *n* = 23. ^b)^Aerobic training group, *n* = 23, control group, *n* = 30, ^c)^Shirom-Melamed Burnout Questionnaire, ^d)^Hospital Anxiety and Depression Scale

### Cognitive function

Group means and standard deviations on domain-specific and global cognitive function, including statistics, for both groups, are presented in Table 3. There were no significant differences between the groups at baseline in domain-specific or global cognitive function. In domain-specific cognitive function, a significant time by group interaction was found for episodic memory, in which the aerobic training group significantly improved in performance more so than the controls. Detailed analysis of the episodic memory test showed a significant time by group interaction in list learning [F(1,53) = 4.20, *p* = 0.04, η_p_^2^ = 0.07], reflecting more robust learning. No other statistically significant differences were observed (Table 3).

**Table 3:** Group means and standard deviations on domain-specific cognitive functions and global cognitive function, in the aerobic training group and the control group before and after the intervention

Variable	Aerobic training group (*n* = 24)		Control group (*n* = 32)		Group x Time		
	T1	T2	T1	T2	F	*p-value*	η_p_^2^
Global cognitive function					F(1,54) = 2.62	0.11	0.05
Domain-specific cognitive function							
Executive function					F(1,54) = 0.45	0.48	0.01
Letter memory running span	2.52 (1.89)	1.97 (1.61)	2.40 (1.89)	2.65 (1.61)			
3-back	20.22 (7.10)	23.64 (6.62)	23.56 (7.10)	25.54 (6.62)			
Inhibition cost	30.72 (11.63)	28.66 (9.08)	27.49 (11.62)	23.48 (9.08)			
Shift Cost	50.74 (22.98)	42.52 (20.44)	43.39 (22.97)	38.23 (20.44)			
Working memory					F(1,54) = 1.80	0.18	0.03
Digit Span Forwards	6.74 (2.01)	6.72 (1.96)	7.42 (2.01)	7.18 (1.96)			
Digit Span Backwards	6.26 (1.84)	6.42 (1.82)	6.15 (1.84)	6.63 (1.82)			
Letter-number sequencing	9.38 (2.22)	10.31 (2.51)	10.65 (2.22)	10.36 (2.50)			
Episodic memory					F(1,54) = 6.11	0.02	0.10
Recall of concrete nouns	50.25 (8.66)	55.79 (9.52)	54.13 (8.65)	53.52 (9.52)			
Perceptual speed					F(1,54) = 1.77	0.19	0.03
Digit symbol	50.26 (11.18)	56.22 (12.19)	55.71 (11.18)	59.62 (12.19)			
Reasoning ability					F(1,54) = 0.42	0.52	0.01
Raven’s matrices	7.46 (2.34)	6.72 (2.37)	7.47 (2.34)	6.27 (2.36)			

Interaction effects statistics are based on z scores

### Psychological variables

No significant time by group interaction effects were observed in any of the psychological variables, see Table 2 for mean values in burnout, anxiety and depression at T1 and T2 respectively.

### Association between improved cognition and improved aerobic capacity

When correlating changes in episodic memory to aerobic capacity variables (VO_2max_ and HRR), no significant correlations were observed (*p* > 0.05).

When comparing subgroups of those who improved (*n* = 12) or did not improve (*n* = 11) in episodic memory after intervention we found a tendency to a larger improvement in HRR in those who improved (*p* = 0.06). There were no significant differences between the subgroups in VO_2max_.

### Hippocampus volume

Results showed no indication of significant change in hippocampus volume following aerobic training. In the aerobic training group mean volume change was −71.2 mm^3^, with a range from −272.3 mm^3^ to +111.5 mm^3^. Two participants in the aerobic group increased and four decreased volume. In the control group, mean volume change was 47.2 mm^3^, with a range from −269.6 mm^3^ to +387.5 mm^3^, eight participants decreased and four increased hippocampus volume.

## Discussion

In this randomized controlled study, we examined if adding a 12-week aerobic training program to patients with ED enrolled in an MMR program facilitated cognitive performance and reduced levels of burnout, depression and anxiety more so than treatment as usual. Our results partially confirmed our hypothesis and showed that the aerobic training group significantly improved in VO_2max_ and in episodic memory performance, as indicated by a medium effect size for this domain (η_p_^2^ = 0.1). No significant group differences where shown in levels of burnout, depression and anxiety, which improved equally in both groups.

The observed improved performance in episodic memory for the patients in the aerobic training group may be of particular interest for this patient group as they improved their ability to retrieve words consistently across trials, which may reflect improved binding of information in episodic memory. Previous studies have shown that patients with ED may experience cognitive impairments [34], which may be only partly reversible through treatment, and still impaired at two-year follow-up [2]. Physical activity is generally considered to have an effect on several cognitive domains, with largest effects in executive functions [35]. A relationship between improved aerobic capacity and improved executive functions has also been shown in males with occupational burnout [14]. Hence, the present findings offer a valuable addition to the literature by showing that adding an aerobic exercise training program within an MMR program of ED may also improve episodic memory functions. There was no significant association between improved oxygen uptake and increased episodic memory performance, which has previously been shown in sedentary healthy adults [36]. However, there was a tendency for a larger improvement in HRR in the aerobic training group for those who improved most in episodic memory, which may be related to the regulation of the autonomic nervous system (ANS) and increased parasympathetic reactivation after exercise. A relationship between HRR, as an index of ANS function, and cognitive functioning has been reported in cardiovascular disease patients [37]. Low heart rate variability (HRV) has been shown in patients with ED, reflecting reduced parasympathetic activity as a consequence of long-term stress exposure [38]. In the same study, they also found that physical activity was related with improved HRV [38]. Thus, we suggest that aerobic training may be of importance in treatment for patients with ED to improve episodic memory performance with a feasible link to the ANS.

Further, in studies of sedentary adults it has been suggested that increased oxygen uptake may be accompanied with increased hippocampus volume [29]. Although limited by only six participants, hippocampus volume did not consequently appear to increase following aerobic exercise for this patient group. It is of importance that future studies investigate this in more detail and disentangle possible neural mechanisms that may account for improved cognitive performance following aerobic exercise in patients with ED.

Despite the addition of aerobic training in our study, no significant differences could be detected in the psychological variables in the intervention group compared to the control group. Previous studies have shown that patients with ED participating in MMR where information on physical activity recommendation are included, also increase their levels of self-reported exercise [39]. Moreover, Lindegård et al. [40] compared patients with ED who complied or not with physical activity recommendations and observed no differences between the groups in levels of burnout, depression and anxiety during a 12-months MMR intervention where all groups improved. However, at 6 months following the end of the intervention the compliers reported significantly lower levels of burnout and depression compared to non-compliers. This is in line with our study, where all the participants took part in MMR during 24 weeks, and in which both groups showed a general improvement in psychological variables. A long-term follow-up will be of interest to specifically address this issue. Also, additional support for MMR to improve psychological health in patients with ED has previously been provided [4]. Based on our data we further support that MMR works well for people participating in rehabilitation, and that the addition of aerobic exercise may influence cognitive functions, in particular episodic memory.

It should be noted that only 51% participated throughout the training period and attendance rate was moderate and just below 60% of planned training sessions. It is possible that greater effects on other cognitive functions e.g. executive performance, would have occurred if the patients had participated in all training sessions or if the intervention had lasted for a longer time period. However, in this study we demonstrated an effect on episodic memory. Increased attendance rate is desirable and dose-response should also be evaluated in future research. Notably, the majority of the patients in the study who actually started aerobic training also managed to perform the exercise at a moderate-vigorous level. Thus, from a clinical perspective, we have shown that it is possible to perform aerobic exercise at a moderate-vigorous intensity in this patient group. Vigorous physical activity and high cardiorespiratory fitness has been shown to have an important role in improving mental health [41], burnout [42] and cardio-metabolic risk factors, especially for individuals with high levels of perceived stress [43]. In this study, individual PAP was one of the components in the MMR, with recommendations to perform physical activity of moderate to high intensity. It is likely that PAP was not providing sufficient support for patients with ED to reach the desired exercise intensity to get effect on fitness, as the control group did not increase their VO_2max_. PAP has previously been shown to increase self-reported level of physical activity in primary care, but it has also been shown that some patients are not following the prescription [44]. Thus, for this patient group we suggest that it is important to provide support and guidance regarding the level of exercise intensity to gain the expected improvements in aerobic capacity.

The present study has some limitations that should be mentioned. The power calculation showed that we needed at least 30 patients in each group to obtain 80% statistical power. We did not fully reach that target. The high dropout rate is likely due to the additional burden from the supplementary assessments and training in combination with efforts in their regular MMR. The main problem for patients with ED is an increased fatigability and difficulties in balancing activities with recuperation. Most patients have also been exposed to a multitude of stressors before falling ill [45]. The MMR in the present study aims at learning to achieve a better balance in life, to set limits, and to prioritize activities. Hence, this may have counteracted fulfilling participation in the study. Obstacles to include additional exercise sessions in MMR was also confirmed in a previous study in which only 21% of patients with ED, who participated in MMR, chose to voluntary participate in an 18-week coached exercise program [39]. Another reason for the high dropout rate may be due to the training method and intensity. We think that the opportunity to choose training method could probably motivate for increased attendance. The intensity of the exercise is probably the most important factor for gaining cardiovascular fitness effects that also improve cognitive function [10].

On the other hand, a major strength of the study was the randomized controlled design with strict recruitment of patients with ED in a clinical setting. The results are important for implementing moderate-vigorous aerobic training in the clinic. One major challenge will be how to best implement aerobic exercise in the rehabilitation program to make it possible for most of the patients to adhere to the training. The timing of different activities in MMR will probably be of great importance for the treatment outcome. We speculate that a greater compliance might have been reached if the training had not been carried out at the same time as the extensive MMR program. Supervised exercise, by e.g. a physiotherapist, could also be of importance to reduce dropout rates [46]. In the future, it would be interesting to get experience from co-design research, in which patients in collaboration with health care professionals design a rehabilitation program with a suitable set-up [47].

## Conclusions

In conclusion, this study shows for the first time that aerobic training may be effective to improve episodic memory in patients with ED, and may inform clinical practise to support exercise at a moderate-vigorous intensity.

## Abbreviations

ANS: Autonomic nervous system
BMI: Body mass index
CBT: Cognitive behavioural therapy
ED: Exhaustion disorder
HAD: Hospital anxiety and depression scale
HRR: Heart rate recovery
HRV: Heart rate variability.
MMR: Multimodal rehabilitation
PAP: Physical activity on prescription
RECO: Rehabilitation for improved cognition
RPE: Ratings of percieved exertion
SMBQ: Shirom-Melamed burnout questionnaire
sMRI: Structural magnetic resonance imagining
Vo2max: Maximal oxygen uptake
